# Racemic 9,10-dimeth­oxy-3-methyl-6-phenyl-7,7a-dihydro­benzo[*b*]benzo[4,5]isothia­zolo[2,3-*d*][1,4]diazepine 12,12-dioxide

**DOI:** 10.1107/S1600536811004983

**Published:** 2011-02-23

**Authors:** Jatinder P. Bassin, Virender P. Shah, Lee Martin, Peter N. Horton

**Affiliations:** a School of Pharmacy, University of Hertfordshire, College Lane, Hatfield AL10 9AB, England; b School of Chemistry, University of Southampton, Highfield, Southampton SO17 1BJ, England

## Abstract

There are two molecules in the asymmetric unit of the title compound, C_24_H_22_N_2_O_4_S. The conformation of the seven-membered ring is twisted boat for both molecules. The molecule is chiral, but crystal symmetry generates a recemate. The crystal packing is stabilized by weak intermolecular C—H⋯O hydrogen bonds.

## Related literature

For related structures, see: Zia-ul-Haq *et al.* (2007 ▶); Boudina *et al.* (2007 ▶); Doubia *et al.* (2007 ▶); Sanudo *et al.* (2009 ▶); Spencer *et al.* (2009 ▶); Swamy *et al.* (2008 ▶). For the psychotropic properties of aptaza­pine [systematic name: 2-methyl-1,3,4,14b-tetra­hydro-2*H*,10*H*-pyrazino­[1,2-*a*]pyrrolo­[2,1-*c*][1,4]benzodiazepine] and bretazenil [systematic name: *t*-butyl-8-bromo-11,12,13,13a-tetra­hydro-9-oxo-9*H*-imidazo(1,5-*a*)pyrrolo­(2,1-*c*)(1,4)benzodiazepine-1-carboxyl­ate], see: Silvestri *et al.* (1994 ▶); Landquist (1984 ▶); Insuasty *et al.* (2008 ▶); Bennamane *et al.* (2008 ▶); Schutz (1982 ▶). For the bioactivity of benzodiazepines, see: Constanzo *et al.* (1990 ▶); Kelly *et al.* (1997 ▶). For the effect on the bioactivity of fusing different heterocyclic rings to the 1,4- and 1,5-benzodiazepine system, see: Chimirri *et al.* (1993**a* ▶,b* ▶). For the synthesis of the title compound, see: Bassin *et al.* (2000 ▶).
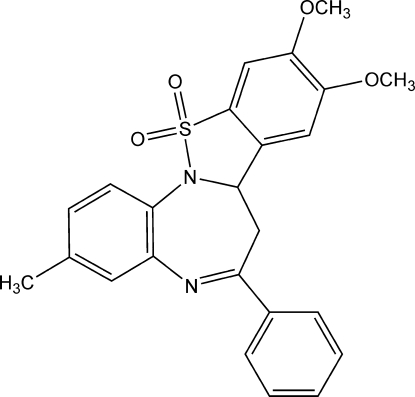


## Experimental

### 

#### Crystal data

C_24_H_22_N_2_O_4_S
*M* *_r_* = 434.51
Triclinic, 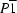

*a* = 11.9007 (4) Å
*b* = 12.8521 (4) Å
*c* = 14.6202 (5) Å
α = 109.852 (2)°
β = 90.743 (2)°
γ = 92.775 (2)°
*V* = 2099.76 (12) Å^3^
*Z* = 4
Mo *K*α radiation
μ = 0.19 mm^−1^
*T* = 120 K
0.50 × 0.30 × 0.10 mm

#### Data collection

Bruker–Nonius KappaCCD diffractometer
Absorption correction: multi-scan (*SORTAV*; Blessing, 1997 ▶) *T*_min_ = 0.912, *T*_max_ = 0.981
31054 measured reflections
7393 independent reflections
5463 reflections with *I* > 2σ(*I*)
*R*_int_ = 0.060

#### Refinement

*R*[*F* ^2^ > 2σ(*F* ^2^)] = 0.041
*wR*(*F* ^2^) = 0.106
*S* = 1.01
7393 reflections
566 parameters
H-atom parameters constrained
Δρ_max_ = 0.29 e Å^−3^
Δρ_min_ = −0.46 e Å^−3^

### 

Data collection: *COLLECT* (Hooft, 1998 ▶); cell refinement: *DENZO* (Otwinowski & Minor, 1997 ▶); data reduction: *DENZO*, *COLLECT* and *maXus* (Mackay *et al.*, 1999 ▶); program(s) used to solve structure: *SHELXS97* (Sheldrick, 2008 ▶); program(s) used to refine structure: *SHELXL97* (Sheldrick, 2008 ▶); molecular graphics: *CAMERON* (Watkin, *et al.*, 1993 ▶); software used to prepare material for publication: *WinGX* (Farrugia, 1999 ▶).

## Supplementary Material

Crystal structure: contains datablocks I, global. DOI: 10.1107/S1600536811004983/rk2256sup1.cif

Structure factors: contains datablocks I. DOI: 10.1107/S1600536811004983/rk2256Isup2.hkl

Additional supplementary materials:  crystallographic information; 3D view; checkCIF report

## Figures and Tables

**Table 1:** Hydrogen-bond geometry (Å, °)

*D*—H⋯*A*	*D*—H	H⋯*A*	*D*⋯*A*	*D*—H⋯*A*
N1—H1*A*⋯Cl1	0.89	2.44	3.303 (3)	162
N1—H1*B*⋯Cl1^i^	0.89	2.36	3.236 (2)	170
N1—H1*C*⋯O1^ii^	0.89	2.05	2.901 (4)	159
O1—H1*H*⋯Cl1	0.85	2.45	3.290 (3)	170
O1—H1*I*⋯Cl1^iii^	0.85	2.39	3.228 (2)	170

Symmetry codes: (i) 
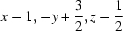
; (ii) 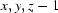; (iii) 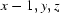.

**Fig. 1.**
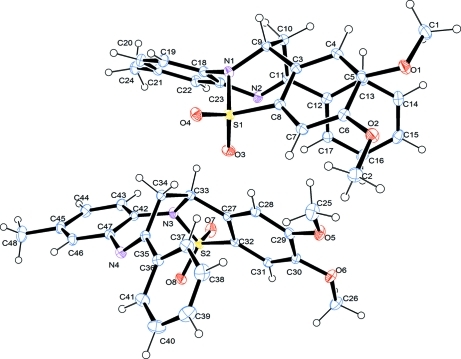
The molecular structure of the title compound, showing the atom labeling scheme. Displacement ellipsoids are drawn at the 30% probability level. H atoms are presented as a small spheres of arbitrary radius.

## supplementary crystallographic information

### Comment

In recent years tetracyclic benzodiazepines have received a great deal of attention due to the psychotropic properties of such compounds as aptazapine and bretazenil (Silvestri *et al.*, 1994; Landquist, 1984; Insuasty *et al.*, 2008; Bennamane *et al.*, 2008; Schutz, 1982). A number of structures of benzodiazepines have been reported: Zia-ul-Haq *et al.*, (2007); Boudina *et al.*, (2007); Doubia *et al.*, (2007); Sanudo *et al.*, (2009); Spencer *et al.*, (2009); Swamy *et al.*, (2008). Benzodiazepines containing heterocycles fused to the seven-membered ring have shown important and varied bioactivities (Constanzo *et al.*, 1990; Kelly *et al.*, 1997). It has been demonstrated that by fusing different heterocyclic rings to the 1,4- and 1,5-benzodiazepine system enhances the biological activity of these compounds (Chimirri *et al.*, 1993*a*,*b*). We previously reported the synthesis of a new heterocyclic ring system dihydrobenzo[*b*]benzo[4,5]isothiazolo[2,3-*d*][1,4]diazepine 12,12-dioxide (Bassin *et al.*, 2000). As, an extension of this work we report the crystal structure of the enantiomeric mixture of 9,10-dimethoxy-3-methyl-6-phenyl-7,7*a*-dihydrobenzo[*b*] benzo[4,5]isothiazolo [2,3-*d*][1,4]diazepine 12,12-dioxide.

### Experimental

The title compound was synthesised following a modified procedure (Bassin *et al.*, 2000) by refluxing (*E*)-4,5-dimethoxy-2-(3-oxo-3-phenylprop-1-en-1-yl)benzene-1-sulfonyl chloride with 3,4-diaminotoluene in ethanol for 1 hour. The reaction mixture was allowed to cool to room temperature the resulting precipitated product was filtered under suction and thoroughly washed with aqueous ethanol. The air dried product was re-crystallised from ethanol. A yellow crystalline product (yield: 82%, m.p.: 498 K) was obtained.

### Refinement

H atoms were positioned geometrically [C–H = 0.95Å (for aromatic), 0.98Å (for methyl groups) and 0.99Å (for methylene groups)] and refined using a riding model, with *U*_iso_(H) = 1.2*U*_eq_(C) and 1.5*U*_eq_(C) for methyl groups.

### Crystal data

**Table:**

C_24_H_22_N_2_O_4_S	*Z* = 4
*M**_r_* = 434.51	*F*(000) = 912
Triclinic, *P*1	*D*_x_ = 1.375 Mg m^−^^3^
Hall symbol: -P 1	Melting point: 498 K
*a* = 11.9007 (4) Å	Mo *K*α radiation, λ = 0.71073 Å
*b* = 12.8521 (4) Å	Cell parameters from 25443 reflections
*c* = 14.6202 (5) Å	θ = 2.9–27.5°
α = 109.852 (2)°	µ = 0.19 mm^−^^1^
β = 90.743 (2)°	*T* = 120 K
γ = 92.775 (2)°	Prism, yellow
*V* = 2099.76 (12) Å^3^	0.50 × 0.30 × 0.10 mm

### Data collection

**Table:**

Bruker–Nonius KappaCCD diffractometer	7393 independent reflections
Radiation source: fine-focus sealed tube	5463 reflections with *I* > 2σ(*I*)
graphite	*R*_int_ = 0.060
Detector resolution: 9.091 pixels mm^-1^	θ_max_ = 25.0°, θ_min_ = 3.0°
φ– and ω scans	*h* = −14→14
Absorption correction: multi-scan (*SORTAV*; Blessing, 1997)	*k* = −15→15
*T*_min_ = 0.912, *T*_max_ = 0.981	*l* = −17→17
31054 measured reflections	

### Refinement

**Table:**

Refinement on *F*^2^	Secondary atom site location: difference Fourier map
Least-squares matrix: full	Hydrogen site location: inferred from neighbouring sites
*R*[*F*^2^ > 2σ(*F*^2^)] = 0.041	H-atom parameters constrained
*wR*(*F*^2^) = 0.106	*w* = 1/[σ^2^(*F*_o_^2^) + (0.0595*P*)^2^ + 0.1891*P*] where *P* = (*F*_o_^2^ + 2*F*_c_^2^)/3
*S* = 1.01	(Δ/σ)_max_ < 0.001
7393 reflections	Δρ_max_ = 0.29 e Å^−^^3^
566 parameters	Δρ_min_ = −0.46 e Å^−^^3^
0 restraints	Extinction correction: *SHELXL97* (Sheldrick, 2008), Fc^*^=kFc[1+0.001xFc^2^λ^3^/sin(2θ)]^-1/4^
Primary atom site location: structure-invariant direct methods	Extinction coefficient: 0.0053 (7)

### Special details

**Table:**

Geometry. All s.u.'s (except the s.u. in the dihedral angle between two l.s. planes) are estimated using the full covariance matrix. The cell s.u.'s are taken into account individually in the estimation of s.u.'s in distances, angles and torsion angles; correlations between s.u.'s in cell parameters are only used when they are defined by crystal symmetry. An approximate (isotropic) treatment of cell s.u.'s is used for estimating s.u.'s involving l.s. planes.
Refinement. Refinement of *F*^2^ against ALL reflections. The weighted *R*-factor *wR* and goodness of fit *S* are based on *F*^2^, conventional *R*-factors *R* are based on *F*, with *F* set to zero for negative *F*^2^. The threshold expression of *F*^2^ > 2σ(*F*^2^) is used only for calculating *R*-factors(gt) *etc*. and is not relevant to the choice of reflections for refinement. *R*-factors based on *F*^2^ are statistically about twice as large as those based on *F*, and *R*-factors based on ALL data will be even larger.

### Fractional atomic coordinates and isotropic or equivalent isotropic displacement parameters (Å^2^)

**Table:**

	*x*	*y*	*z*	*U*_iso_*/*U*_eq_	
C1	0.3992 (2)	−0.2363 (2)	0.14280 (18)	0.0360 (6)	
H1A	0.4094	−0.1782	0.2067	0.054*	
H1B	0.4607	−0.2867	0.1329	0.054*	
H1C	0.3272	−0.2778	0.1396	0.054*	
C2	0.42922 (19)	−0.05068 (19)	−0.13723 (16)	0.0311 (5)	
H2A	0.3605	−0.0683	−0.1784	0.047*	
H2B	0.4933	−0.0824	−0.1766	0.047*	
H2C	0.4423	0.0299	−0.1089	0.047*	
C3	0.16980 (16)	0.00163 (15)	0.14164 (14)	0.0188 (4)	
C4	0.24041 (17)	−0.08172 (16)	0.14182 (14)	0.0217 (5)	
H4	0.2314	−0.1188	0.1877	0.026*	
C5	0.32354 (17)	−0.10949 (16)	0.07437 (15)	0.0225 (5)	
C6	0.33481 (17)	−0.05692 (16)	0.00370 (14)	0.0215 (5)	
C7	0.26602 (17)	0.02657 (16)	0.00462 (14)	0.0222 (5)	
H7	0.2738	0.0637	−0.0413	0.027*	
C8	0.18439 (16)	0.05466 (15)	0.07553 (14)	0.0197 (4)	
C9	0.07022 (16)	0.03569 (16)	0.20530 (14)	0.0195 (4)	
H9	0.0141	−0.0289	0.1893	0.023*	
C10	0.09793 (17)	0.07586 (16)	0.31449 (14)	0.0211 (4)	
H10A	0.0271	0.0850	0.3505	0.025*	
H10B	0.1404	0.0195	0.3303	0.025*	
C11	0.16693 (16)	0.18492 (16)	0.34651 (13)	0.0194 (4)	
C12	0.29050 (17)	0.18973 (16)	0.36576 (14)	0.0210 (5)	
C13	0.34603 (18)	0.10086 (17)	0.37451 (15)	0.0265 (5)	
H13	0.3046	0.0338	0.3690	0.032*	
C14	0.46215 (18)	0.10953 (19)	0.39138 (16)	0.0306 (5)	
H14	0.4992	0.0483	0.3976	0.037*	
C15	0.52371 (18)	0.20556 (19)	0.39914 (15)	0.0317 (5)	
H15	0.6030	0.2107	0.4100	0.038*	
C16	0.46915 (18)	0.2947 (2)	0.39095 (17)	0.0334 (6)	
H16	0.5111	0.3614	0.3962	0.040*	
C17	0.35396 (17)	0.28733 (18)	0.37510 (15)	0.0272 (5)	
H17	0.3173	0.3495	0.3705	0.033*	
C18	−0.04226 (16)	0.20694 (16)	0.24328 (14)	0.0207 (5)	
C19	−0.15279 (17)	0.21991 (17)	0.22056 (15)	0.0250 (5)	
H19	−0.1860	0.1735	0.1601	0.030*	
C20	−0.21585 (17)	0.29909 (17)	0.28400 (15)	0.0259 (5)	
H20	−0.2914	0.3066	0.2665	0.031*	
C21	−0.16983 (17)	0.36800 (16)	0.37326 (15)	0.0235 (5)	
C22	−0.05783 (17)	0.35632 (16)	0.39450 (15)	0.0235 (5)	
H22	−0.0245	0.4042	0.4543	0.028*	
C23	0.00699 (17)	0.27780 (16)	0.33205 (15)	0.0213 (5)	
C24	−0.23848 (18)	0.45185 (18)	0.44470 (16)	0.0308 (5)	
H24A	−0.2971	0.4754	0.4095	0.046*	
H24B	−0.1895	0.5162	0.4820	0.046*	
H24C	−0.2737	0.4185	0.4892	0.046*	
N1	0.02092 (13)	0.12406 (13)	0.17640 (11)	0.0204 (4)	
N2	0.12208 (13)	0.27710 (13)	0.35685 (12)	0.0219 (4)	
O1	0.40000 (12)	−0.18679 (11)	0.06850 (10)	0.0290 (4)	
O2	0.41702 (12)	−0.09601 (12)	−0.06133 (10)	0.0282 (3)	
O3	0.13467 (12)	0.26485 (11)	0.12453 (10)	0.0278 (4)	
O4	0.01497 (12)	0.12890 (11)	0.00291 (10)	0.0265 (3)	
S1	0.08537 (4)	0.15358 (4)	0.08842 (4)	0.02013 (14)	
C25	0.4183 (2)	0.30217 (18)	0.13538 (18)	0.0388 (6)	
H25A	0.3708	0.2635	0.1693	0.058*	
H25B	0.4746	0.2526	0.0987	0.058*	
H25C	0.3714	0.3246	0.0905	0.058*	
C26	0.64950 (18)	0.67805 (19)	0.35264 (17)	0.0348 (6)	
H26A	0.6307	0.7317	0.3214	0.052*	
H26B	0.7296	0.6639	0.3453	0.052*	
H26C	0.6332	0.7079	0.4220	0.052*	
C27	0.23523 (16)	0.57054 (16)	0.29537 (14)	0.0188 (4)	
C28	0.29256 (17)	0.47623 (16)	0.24852 (14)	0.0218 (5)	
H28	0.2523	0.4079	0.2147	0.026*	
C29	0.40916 (17)	0.48312 (16)	0.25179 (14)	0.0233 (5)	
C30	0.46967 (16)	0.58300 (16)	0.30635 (14)	0.0215 (5)	
C31	0.41321 (17)	0.67598 (16)	0.35242 (14)	0.0210 (5)	
H31	0.4527	0.7439	0.3885	0.025*	
C32	0.29588 (16)	0.66768 (16)	0.34460 (14)	0.0192 (4)	
C33	0.10869 (17)	0.57821 (16)	0.30258 (14)	0.0205 (4)	
H33	0.0811	0.5345	0.3437	0.025*	
C34	0.04001 (16)	0.53834 (16)	0.20675 (14)	0.0201 (4)	
H34A	0.0568	0.4608	0.1700	0.024*	
H34B	−0.0411	0.5395	0.2210	0.024*	
C35	0.06480 (17)	0.60900 (16)	0.14439 (14)	0.0213 (5)	
C36	0.13310 (17)	0.56838 (16)	0.05648 (14)	0.0224 (5)	
C37	0.17889 (19)	0.46524 (18)	0.02645 (16)	0.0309 (5)	
H37	0.1614	0.4149	0.0598	0.037*	
C38	0.2498 (2)	0.4349 (2)	−0.05163 (17)	0.0401 (6)	
H38	0.2808	0.3642	−0.0712	0.048*	
C39	0.2754 (2)	0.5066 (2)	−0.10083 (17)	0.0419 (6)	
H39	0.3262	0.4868	−0.1528	0.050*	
C40	0.2270 (2)	0.6071 (2)	−0.07446 (17)	0.0416 (6)	
H40	0.2429	0.6558	−0.1096	0.050*	
C41	0.1557 (2)	0.63781 (19)	0.00233 (16)	0.0320 (5)	
H41	0.1215	0.7068	0.0187	0.038*	
C42	−0.00310 (16)	0.75312 (16)	0.34128 (15)	0.0209 (5)	
C43	−0.05867 (18)	0.81701 (18)	0.42232 (16)	0.0274 (5)	
H43	−0.0384	0.8152	0.4848	0.033*	
C44	−0.14299 (18)	0.88309 (18)	0.41306 (17)	0.0310 (5)	
H44	−0.1793	0.9274	0.4694	0.037*	
C45	−0.17537 (17)	0.88550 (17)	0.32215 (17)	0.0284 (5)	
C46	−0.11840 (17)	0.82177 (17)	0.24161 (16)	0.0263 (5)	
H46	−0.1396	0.8231	0.1792	0.032*	
C47	−0.03145 (17)	0.75615 (16)	0.24917 (15)	0.0222 (5)	
C48	−0.2672 (2)	0.9570 (2)	0.3111 (2)	0.0432 (6)	
H48A	−0.2339	1.0214	0.2976	0.065*	
H48B	−0.3089	0.9822	0.3714	0.065*	
H48C	−0.3186	0.9142	0.2571	0.065*	
N3	0.09285 (14)	0.69706 (13)	0.35612 (12)	0.0215 (4)	
N4	0.02928 (14)	0.70698 (14)	0.16533 (12)	0.0235 (4)	
O5	0.47410 (12)	0.39866 (11)	0.20519 (11)	0.0310 (4)	
O6	0.58380 (11)	0.57683 (12)	0.30791 (10)	0.0289 (4)	
O7	0.21847 (12)	0.81169 (12)	0.50136 (10)	0.0318 (4)	
O8	0.21966 (12)	0.86010 (11)	0.35488 (11)	0.0325 (4)	
S2	0.20873 (4)	0.77439 (4)	0.39734 (4)	0.02169 (14)	

### Atomic displacement parameters (Å^2^)

**Table:**

	*U*^11^	*U*^22^	*U*^33^	*U*^12^	*U*^13^	*U*^23^
C1	0.0384 (14)	0.0364 (13)	0.0432 (15)	0.0115 (11)	0.0032 (11)	0.0250 (12)
C2	0.0318 (13)	0.0387 (13)	0.0250 (13)	0.0040 (10)	0.0060 (10)	0.0130 (10)
C3	0.0219 (11)	0.0144 (10)	0.0171 (11)	−0.0015 (8)	−0.0029 (8)	0.0020 (8)
C4	0.0272 (12)	0.0181 (10)	0.0210 (11)	−0.0001 (9)	−0.0018 (9)	0.0085 (9)
C5	0.0243 (11)	0.0168 (10)	0.0257 (12)	0.0038 (9)	0.0001 (9)	0.0058 (9)
C6	0.0221 (11)	0.0213 (11)	0.0181 (11)	0.0007 (9)	0.0001 (9)	0.0028 (9)
C7	0.0250 (11)	0.0215 (11)	0.0212 (11)	0.0003 (9)	−0.0003 (9)	0.0089 (9)
C8	0.0219 (11)	0.0154 (10)	0.0197 (11)	−0.0015 (8)	−0.0028 (9)	0.0038 (8)
C9	0.0208 (11)	0.0170 (10)	0.0205 (11)	0.0008 (8)	−0.0005 (8)	0.0061 (8)
C10	0.0222 (11)	0.0209 (11)	0.0195 (11)	0.0018 (9)	0.0003 (8)	0.0061 (9)
C11	0.0229 (11)	0.0201 (11)	0.0136 (10)	0.0013 (9)	0.0003 (8)	0.0038 (8)
C12	0.0238 (11)	0.0241 (11)	0.0129 (10)	0.0015 (9)	0.0005 (8)	0.0034 (8)
C13	0.0261 (12)	0.0243 (12)	0.0271 (12)	0.0036 (9)	0.0006 (9)	0.0058 (9)
C14	0.0295 (13)	0.0343 (13)	0.0266 (13)	0.0117 (11)	−0.0012 (10)	0.0074 (10)
C15	0.0224 (12)	0.0484 (15)	0.0234 (13)	0.0033 (11)	−0.0003 (9)	0.0110 (11)
C16	0.0235 (12)	0.0401 (14)	0.0390 (14)	−0.0061 (11)	−0.0050 (10)	0.0180 (11)
C17	0.0249 (12)	0.0279 (12)	0.0306 (13)	0.0016 (10)	−0.0031 (9)	0.0123 (10)
C18	0.0211 (11)	0.0182 (10)	0.0224 (11)	0.0027 (9)	0.0023 (9)	0.0063 (9)
C19	0.0231 (11)	0.0267 (11)	0.0224 (12)	0.0012 (9)	−0.0028 (9)	0.0049 (9)
C20	0.0178 (11)	0.0305 (12)	0.0290 (13)	0.0039 (9)	−0.0006 (9)	0.0095 (10)
C21	0.0239 (11)	0.0202 (11)	0.0273 (12)	0.0037 (9)	0.0021 (9)	0.0090 (9)
C22	0.0248 (11)	0.0200 (11)	0.0227 (12)	0.0008 (9)	−0.0036 (9)	0.0037 (9)
C23	0.0215 (11)	0.0186 (10)	0.0235 (11)	−0.0001 (9)	−0.0010 (9)	0.0069 (9)
C24	0.0274 (12)	0.0310 (12)	0.0320 (13)	0.0075 (10)	0.0024 (10)	0.0074 (10)
N1	0.0225 (9)	0.0190 (9)	0.0200 (9)	0.0036 (7)	0.0003 (7)	0.0067 (7)
N2	0.0194 (9)	0.0219 (9)	0.0214 (10)	0.0016 (7)	−0.0016 (7)	0.0036 (7)
O1	0.0336 (9)	0.0255 (8)	0.0316 (9)	0.0116 (7)	0.0054 (7)	0.0131 (7)
O2	0.0317 (8)	0.0301 (8)	0.0250 (8)	0.0102 (7)	0.0094 (7)	0.0110 (7)
O3	0.0316 (8)	0.0169 (7)	0.0333 (9)	−0.0017 (6)	0.0000 (7)	0.0069 (6)
O4	0.0308 (8)	0.0269 (8)	0.0217 (8)	0.0024 (7)	−0.0058 (6)	0.0084 (6)
S1	0.0222 (3)	0.0166 (3)	0.0209 (3)	0.0006 (2)	−0.0011 (2)	0.0056 (2)
C25	0.0392 (14)	0.0256 (12)	0.0386 (14)	0.0070 (11)	−0.0016 (11)	−0.0064 (11)
C26	0.0240 (12)	0.0359 (13)	0.0395 (14)	−0.0033 (10)	0.0003 (10)	0.0072 (11)
C27	0.0199 (10)	0.0196 (11)	0.0193 (11)	0.0006 (9)	−0.0005 (8)	0.0098 (9)
C28	0.0230 (11)	0.0168 (10)	0.0243 (12)	0.0010 (9)	−0.0004 (9)	0.0053 (9)
C29	0.0280 (12)	0.0202 (11)	0.0213 (12)	0.0077 (9)	0.0028 (9)	0.0057 (9)
C30	0.0188 (11)	0.0258 (11)	0.0201 (11)	0.0022 (9)	0.0005 (8)	0.0080 (9)
C31	0.0238 (11)	0.0178 (10)	0.0203 (11)	−0.0015 (9)	−0.0015 (9)	0.0057 (9)
C32	0.0231 (11)	0.0172 (10)	0.0185 (11)	0.0022 (9)	0.0019 (8)	0.0076 (8)
C33	0.0242 (11)	0.0162 (10)	0.0210 (11)	0.0027 (9)	0.0017 (9)	0.0060 (8)
C34	0.0184 (10)	0.0183 (10)	0.0213 (11)	0.0017 (8)	0.0008 (8)	0.0037 (9)
C35	0.0207 (11)	0.0218 (11)	0.0193 (11)	−0.0007 (9)	−0.0038 (8)	0.0046 (9)
C36	0.0243 (11)	0.0217 (11)	0.0187 (11)	0.0002 (9)	−0.0014 (9)	0.0039 (9)
C37	0.0371 (13)	0.0298 (12)	0.0243 (13)	0.0038 (10)	0.0039 (10)	0.0068 (10)
C38	0.0436 (15)	0.0391 (14)	0.0313 (14)	0.0121 (12)	0.0101 (11)	0.0024 (11)
C39	0.0426 (15)	0.0534 (17)	0.0231 (13)	0.0006 (13)	0.0106 (11)	0.0046 (12)
C40	0.0554 (16)	0.0442 (15)	0.0267 (14)	−0.0024 (13)	0.0079 (12)	0.0145 (12)
C41	0.0407 (14)	0.0310 (12)	0.0237 (13)	0.0012 (11)	0.0012 (10)	0.0086 (10)
C42	0.0183 (10)	0.0191 (10)	0.0247 (12)	0.0022 (9)	0.0019 (9)	0.0065 (9)
C43	0.0256 (12)	0.0298 (12)	0.0247 (12)	0.0041 (10)	0.0021 (9)	0.0060 (10)
C44	0.0263 (12)	0.0283 (12)	0.0329 (14)	0.0087 (10)	0.0076 (10)	0.0020 (10)
C45	0.0227 (11)	0.0209 (11)	0.0386 (14)	0.0036 (9)	−0.0028 (10)	0.0059 (10)
C46	0.0257 (12)	0.0221 (11)	0.0301 (13)	0.0008 (9)	−0.0056 (9)	0.0078 (10)
C47	0.0223 (11)	0.0177 (10)	0.0248 (12)	0.0001 (9)	0.0004 (9)	0.0053 (9)
C48	0.0348 (14)	0.0366 (14)	0.0522 (17)	0.0131 (11)	−0.0065 (12)	0.0059 (12)
N3	0.0223 (9)	0.0180 (9)	0.0219 (9)	0.0034 (7)	−0.0019 (7)	0.0037 (7)
N4	0.0261 (10)	0.0224 (10)	0.0214 (10)	0.0021 (8)	−0.0008 (8)	0.0066 (8)
O5	0.0253 (8)	0.0238 (8)	0.0367 (9)	0.0079 (7)	0.0020 (7)	0.0002 (7)
O6	0.0196 (8)	0.0302 (8)	0.0313 (9)	0.0003 (6)	0.0004 (6)	0.0032 (7)
O7	0.0326 (9)	0.0336 (9)	0.0205 (8)	0.0070 (7)	−0.0052 (6)	−0.0024 (7)
O8	0.0325 (9)	0.0213 (8)	0.0480 (10)	0.0031 (7)	−0.0025 (7)	0.0172 (7)
S2	0.0230 (3)	0.0175 (3)	0.0231 (3)	0.0027 (2)	−0.0020 (2)	0.0048 (2)

### Geometric parameters (Å, °)

**Table:**

C1—O1	1.433 (2)	C25—O5	1.437 (3)
C1—H1A	0.9800	C25—H25A	0.9800
C1—H1B	0.9800	C25—H25B	0.9800
C1—H1C	0.9800	C25—H25C	0.9800
C2—O2	1.424 (2)	C26—O6	1.429 (3)
C2—H2A	0.9800	C26—H26A	0.9800
C2—H2B	0.9800	C26—H26B	0.9800
C2—H2C	0.9800	C26—H26C	0.9800
C3—C8	1.367 (3)	C27—C32	1.373 (3)
C3—C4	1.394 (3)	C27—C28	1.390 (3)
C3—C9	1.505 (3)	C27—C33	1.517 (3)
C4—C5	1.378 (3)	C28—C29	1.385 (3)
C4—H4	0.9500	C28—H28	0.9500
C5—O1	1.362 (2)	C29—O5	1.354 (2)
C5—C6	1.418 (3)	C29—C30	1.415 (3)
C6—O2	1.364 (2)	C30—O6	1.365 (2)
C6—C7	1.377 (3)	C30—C31	1.370 (3)
C7—C8	1.398 (3)	C31—C32	1.395 (3)
C7—H7	0.9500	C31—H31	0.9500
C8—S1	1.740 (2)	C32—S2	1.730 (2)
C9—N1	1.482 (2)	C33—N3	1.484 (3)
C9—C10	1.528 (3)	C33—C34	1.528 (3)
C9—H9	1.0000	C33—H33	1.0000
C10—C11	1.514 (3)	C34—C35	1.512 (3)
C10—H10A	0.9900	C34—H34A	0.9900
C10—H10B	0.9900	C34—H34B	0.9900
C11—N2	1.286 (2)	C35—N4	1.285 (3)
C11—C12	1.488 (3)	C35—C36	1.482 (3)
C12—C13	1.388 (3)	C36—C37	1.388 (3)
C12—C17	1.397 (3)	C36—C41	1.399 (3)
C13—C14	1.393 (3)	C37—C38	1.386 (3)
C13—H13	0.9500	C37—H37	0.9500
C14—C15	1.374 (3)	C38—C39	1.374 (3)
C14—H14	0.9500	C38—H38	0.9500
C15—C16	1.383 (3)	C39—C40	1.374 (4)
C15—H15	0.9500	C39—H39	0.9500
C16—C17	1.381 (3)	C40—C41	1.376 (3)
C16—H16	0.9500	C40—H40	0.9500
C17—H17	0.9500	C41—H41	0.9500
C18—C19	1.384 (3)	C42—C43	1.388 (3)
C18—C23	1.408 (3)	C42—C47	1.398 (3)
C18—N1	1.430 (3)	C42—N3	1.432 (3)
C19—C20	1.382 (3)	C43—C44	1.380 (3)
C19—H19	0.9500	C43—H43	0.9500
C20—C21	1.392 (3)	C44—C45	1.390 (3)
C20—H20	0.9500	C44—H44	0.9500
C21—C22	1.391 (3)	C45—C46	1.392 (3)
C21—C24	1.504 (3)	C45—C48	1.503 (3)
C22—C23	1.384 (3)	C46—C47	1.393 (3)
C22—H22	0.9500	C46—H46	0.9500
C23—N2	1.413 (3)	C47—N4	1.402 (3)
C24—H24A	0.9800	C48—H48A	0.9800
C24—H24B	0.9800	C48—H48B	0.9800
C24—H24C	0.9800	C48—H48C	0.9800
N1—S1	1.6479 (16)	N3—S2	1.6406 (17)
O3—S1	1.4374 (14)	O7—S2	1.4321 (15)
O4—S1	1.4295 (14)	O8—S2	1.4383 (15)
			
O1—C1—H1A	109.5	O5—C25—H25A	109.5
O1—C1—H1B	109.5	O5—C25—H25B	109.5
H1A—C1—H1B	109.5	H25A—C25—H25B	109.5
O1—C1—H1C	109.5	O5—C25—H25C	109.5
H1A—C1—H1C	109.5	H25A—C25—H25C	109.5
H1B—C1—H1C	109.5	H25B—C25—H25C	109.5
O2—C2—H2A	109.5	O6—C26—H26A	109.5
O2—C2—H2B	109.5	O6—C26—H26B	109.5
H2A—C2—H2B	109.5	H26A—C26—H26B	109.5
O2—C2—H2C	109.5	O6—C26—H26C	109.5
H2A—C2—H2C	109.5	H26A—C26—H26C	109.5
H2B—C2—H2C	109.5	H26B—C26—H26C	109.5
C8—C3—C4	119.80 (19)	C32—C27—C28	119.02 (18)
C8—C3—C9	114.71 (17)	C32—C27—C33	114.27 (17)
C4—C3—C9	125.35 (17)	C28—C27—C33	126.65 (17)
C5—C4—C3	118.94 (18)	C29—C28—C27	119.12 (18)
C5—C4—H4	120.5	C29—C28—H28	120.4
C3—C4—H4	120.5	C27—C28—H28	120.4
O1—C5—C4	124.86 (18)	O5—C29—C28	124.54 (18)
O1—C5—C6	114.49 (17)	O5—C29—C30	114.72 (17)
C4—C5—C6	120.65 (18)	C28—C29—C30	120.74 (18)
O2—C6—C7	125.27 (18)	O6—C30—C31	125.14 (18)
O2—C6—C5	114.49 (17)	O6—C30—C29	114.80 (17)
C7—C6—C5	120.23 (18)	C31—C30—C29	120.06 (18)
C6—C7—C8	117.64 (18)	C30—C31—C32	117.85 (18)
C6—C7—H7	121.2	C30—C31—H31	121.1
C8—C7—H7	121.2	C32—C31—H31	121.1
C3—C8—C7	122.65 (18)	C27—C32—C31	123.09 (18)
C3—C8—S1	111.35 (15)	C27—C32—S2	111.58 (15)
C7—C8—S1	125.92 (15)	C31—C32—S2	125.30 (15)
N1—C9—C3	105.11 (15)	N3—C33—C27	104.51 (15)
N1—C9—C10	111.04 (15)	N3—C33—C34	110.71 (15)
C3—C9—C10	114.78 (16)	C27—C33—C34	116.61 (16)
N1—C9—H9	108.6	N3—C33—H33	108.2
C3—C9—H9	108.6	C27—C33—H33	108.2
C10—C9—H9	108.6	C34—C33—H33	108.2
C11—C10—C9	111.25 (15)	C35—C34—C33	112.47 (16)
C11—C10—H10A	109.4	C35—C34—H34A	109.1
C9—C10—H10A	109.4	C33—C34—H34A	109.1
C11—C10—H10B	109.4	C35—C34—H34B	109.1
C9—C10—H10B	109.4	C33—C34—H34B	109.1
H10A—C10—H10B	108.0	H34A—C34—H34B	107.8
N2—C11—C12	117.02 (17)	N4—C35—C36	116.87 (18)
N2—C11—C10	121.73 (17)	N4—C35—C34	122.00 (18)
C12—C11—C10	121.24 (17)	C36—C35—C34	121.12 (17)
C13—C12—C17	118.26 (19)	C37—C36—C41	118.0 (2)
C13—C12—C11	122.76 (18)	C37—C36—C35	123.05 (18)
C17—C12—C11	118.97 (18)	C41—C36—C35	118.94 (19)
C12—C13—C14	120.3 (2)	C38—C37—C36	120.7 (2)
C12—C13—H13	119.8	C38—C37—H37	119.6
C14—C13—H13	119.8	C36—C37—H37	119.6
C15—C14—C13	120.8 (2)	C39—C38—C37	120.3 (2)
C15—C14—H14	119.6	C39—C38—H38	119.8
C13—C14—H14	119.6	C37—C38—H38	119.8
C14—C15—C16	119.4 (2)	C38—C39—C40	119.6 (2)
C14—C15—H15	120.3	C38—C39—H39	120.2
C16—C15—H15	120.3	C40—C39—H39	120.2
C17—C16—C15	120.3 (2)	C39—C40—C41	120.7 (2)
C17—C16—H16	119.8	C39—C40—H40	119.7
C15—C16—H16	119.8	C41—C40—H40	119.7
C16—C17—C12	120.9 (2)	C40—C41—C36	120.6 (2)
C16—C17—H17	119.5	C40—C41—H41	119.7
C12—C17—H17	119.5	C36—C41—H41	119.7
C19—C18—C23	118.93 (18)	C43—C42—C47	120.06 (19)
C19—C18—N1	120.14 (18)	C43—C42—N3	118.37 (18)
C23—C18—N1	120.92 (17)	C47—C42—N3	121.01 (17)
C20—C19—C18	121.32 (19)	C44—C43—C42	120.7 (2)
C20—C19—H19	119.3	C44—C43—H43	119.6
C18—C19—H19	119.3	C42—C43—H43	119.6
C19—C20—C21	120.70 (19)	C43—C44—C45	120.6 (2)
C19—C20—H20	119.6	C43—C44—H44	119.7
C21—C20—H20	119.6	C45—C44—H44	119.7
C22—C21—C20	117.61 (19)	C44—C45—C46	118.09 (19)
C22—C21—C24	120.97 (18)	C44—C45—C48	121.1 (2)
C20—C21—C24	121.42 (18)	C46—C45—C48	120.8 (2)
C23—C22—C21	122.67 (19)	C45—C46—C47	122.4 (2)
C23—C22—H22	118.7	C45—C46—H46	118.8
C21—C22—H22	118.7	C47—C46—H46	118.8
C22—C23—C18	118.72 (18)	C46—C47—C42	118.05 (19)
C22—C23—N2	118.58 (17)	C46—C47—N4	117.27 (18)
C18—C23—N2	122.55 (18)	C42—C47—N4	124.33 (18)
C21—C24—H24A	109.5	C45—C48—H48A	109.5
C21—C24—H24B	109.5	C45—C48—H48B	109.5
H24A—C24—H24B	109.5	H48A—C48—H48B	109.5
C21—C24—H24C	109.5	C45—C48—H48C	109.5
H24A—C24—H24C	109.5	H48A—C48—H48C	109.5
H24B—C24—H24C	109.5	H48B—C48—H48C	109.5
C18—N1—C9	120.90 (15)	C42—N3—C33	123.39 (16)
C18—N1—S1	118.90 (13)	C42—N3—S2	116.39 (12)
C9—N1—S1	115.15 (13)	C33—N3—S2	115.50 (13)
C11—N2—C23	120.09 (16)	C35—N4—C47	121.94 (17)
C5—O1—C1	117.25 (16)	C29—O5—C25	116.94 (16)
C6—O2—C2	117.44 (16)	C30—O6—C26	117.05 (16)
O4—S1—O3	114.70 (8)	O7—S2—O8	115.28 (9)
O4—S1—N1	112.29 (8)	O7—S2—N3	111.89 (9)
O3—S1—N1	110.02 (8)	O8—S2—N3	110.19 (9)
O4—S1—C8	111.83 (9)	O7—S2—C32	111.60 (9)
O3—S1—C8	112.54 (9)	O8—S2—C32	112.07 (9)
N1—S1—C8	93.60 (9)	N3—S2—C32	93.86 (9)

### Hydrogen-bond geometry (Å, °)

**Table:**

*D*—H···*A*	*D*—H	H···*A*	*D*···*A*	*D*—H···*A*
C2—H2C···O1^i^	0.98	2.59	3.423 (3)	142
C4—H4···O8^ii^	0.95	2.55	3.451 (3)	157
C10—H10B···O8^ii^	0.99	2.43	3.408 (3)	171
C13—H13···O8^ii^	0.95	2.35	3.295 (3)	174
C15—H15···O7^iii^	0.95	2.55	3.428 (3)	153
C25—H25B···O2^i^	0.98	2.35	3.262 (3)	154
C28—H28···O3	0.95	2.27	3.205 (3)	169
C34—H34A···O3	0.99	2.60	3.552 (3)	162
C37—H37···O3	0.95	2.43	3.371 (3)	170
C39—H39···O6^iv^	0.95	2.42	3.350 (3)	167

Symmetry codes: (i) −*x*+1, −*y*, −*z*; (ii) *x*, *y*−1, *z*; (iii) −*x*+1, −*y*+1, −*z*+1; (iv) −*x*+1, −*y*+1, −*z*.
